# The development of an unsupervised hierarchical clustering analysis of dual‐polarization weather surveillance radar observations to assess nocturnal insect abundance and diversity

**DOI:** 10.1002/rse2.270

**Published:** 2022-05-24

**Authors:** Maryna Lukach, Thomas Dally, William Evans, Christopher Hassall, Elizabeth J. Duncan, Lindsay Bennett, Freya I. Addison, William E. Kunin, Jason W. Chapman, Ryan R. Neely

**Affiliations:** ^1^ National Centre for Atmospheric Science and the School of Earth and Environment University of Leeds 71‐75 Clarendon Rd, Woodhouse Leeds LS2 9PH UK; ^2^ School of Biology, Faculty of Biological Sciences University of Leeds Woodhouse Lane Leeds LS2 9JT UK; ^3^ Centre for Ecology and Conservation, and Environment and Sustainability Institute University of Exeter Penryn, Cornwall TR10 9FE UK; ^4^ Department of Entomology, College of Plant Protection Nanjing Agricultural University Nanjing 210095 People's Republic of China

**Keywords:** Abundance, biodiversity, dual‐polarization, insects, moths, nocturnal, weather radar

## Abstract

Contemporary analyses of insect population trends are based, for the most part, on a large body of heterogeneous and short‐term datasets of diurnal species that are representative of limited spatial domains. This makes monitoring changes in insect biomass and biodiversity difficult. What is needed is a method for monitoring that provides a consistent, high‐resolution picture of insect populations through time over large areas during day and night. Here, we explore the use of X‐band weather surveillance radar (WSR) for the study of local insect populations using a high‐quality, multi‐week time series of nocturnal moth light trapping data. Specifically, we test the hypotheses that (i) unsupervised data‐driven classification algorithms can differentiate meteorological and biological phenomena, (ii) the diversity of the classes of bioscatterers are quantitatively related to the diversity of insects as measured on the ground and (iii) insect abundance measured at ground level can be predicted quantitatively based on dual‐polarization Doppler WSR variables. Adapting the quasi‐vertical profile analysis method and data clustering techniques developed for the analysis of hydrometeors, we demonstrate that our bioscatterer classification algorithm successfully differentiates bioscatterers from hydrometeors over a large spatial scale and at high temporal resolutions. Furthermore, our results also show a clear relationship between biological and meteorological scatterers and a link between the abundance and diversity of radar‐based bioscatterer clusters and that of nocturnal aerial insects. Thus, we demonstrate the potential utility of this approach for landscape scale monitoring of biodiversity.

## Graphical abstract

Here, we explore the use of X‐band weather radar for the study of local insect populations using a high‐quality, multi‐week time series of nocturnal moth light trapping data. Specifically, we test the hypotheses that (i) classification algorithms can differentiate meteorological and biological phenomena, (ii) the diversity of the classes of bioscatterers are quantitatively related to the diversity of insects as measured on the ground, and (iii) insect abundance measured at ground level can be predicted quantitatively based on dual‐polarisation Doppler weather radar variables. Adapting the quasi‐vertical profile (QVP) analysis method and data clustering techniques developed for the analysis of hydrometeors, we demonstrate that our bioscatterer classification algorithm successfully differentiates bioscatterers from hydrometeors over a large spatial scale and at high temporal resolutions.

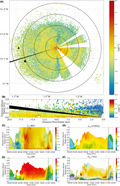

## Introduction

Insect population trends have been the focus of large numbers of research publications over the past decade, with the majority indicating declines (e.g. Hallmann et al., 2017; van Klink et al., 2020; Wagner, 2020). However, the quality of the data available has led to debate over the validity, or at least generalizability, of many of those findings (Desquilbet et al., 2021; Simmons et al., 2019). The field of aeroecology has the potential to contribute a novel and standardized approach to entomological monitoring, but our understanding of how changes in the environment affect aerial insect diversity at the macroscale is still in its infancy (Bauer et al., 2017, 2019; Crossley et al., 2020; Didham et al., 2020; Shamoun‐Baranes et al., 2019; Stepanian et al., 2020). With observations of birds and insects dating back to the first use of radar for tracking aircraft, there is a long tradition of incorporating radar technology into biological studies to observe and quantify airborne animals (Chapman et al., 2002, 2011; Drake & Reynolds, 2012; Hao et al., 2020), and observations from weather surveillance radars (WSRs) have long been thought of as tools that could provide aeroecological observations over large areas (Stepanian et al., 2016; Vaughn, 1985; Westbrook & Eyster, 2017). In addition to measuring hydrometeors (e.g. rain, snow, hail), WSRs continuously monitor the movements of volant animals (i.e. insects, birds and bats) over large spatial and temporal domains with resolution of <1 km and ~5 min. By combining the observations from national‐ and continental‐scale networks, WSRs have the potential to provide unprecedented information about macroecological patterns. Yet this potential has not been fully realized because few rigorous methods exist for separating, identifying and classifying biological information within WSR observations (Gauthreaux & Diehl, 2020). Here, we describe a novel, unsupervized method for extracting information about bioscatterers (i.e. volant biological scatterers) from dual‐polarization Doppler WSRs.

Numerous proof‐of‐concept and observational studies have shown that Doppler WSRs, with and without dual‐polarization, can be used to observe birds and insects and have argued for the potential of WSRs to provide insights into ecology (e.g. Bachmann & Zrnić, 2007; Chapman et al., 2011; Chilson, Bridge, et al., 2012; Chilson, Frick, et al., 2012; Dokter et al., 2011; Drake, 1990; Gauthreaux et al., 2008; Gauthreaux & Belser, 1998; Gourley et al., 2007; Melnikov et al., 2014; Melnikov et al., 2015; Rennie et al., 2010; Russell & Wilson, 1997; Russell & Wilson, 2001; Stepanian et al., 2020; Tielens et al., 2021; Westbrook et al., 2014; Westbrook & Eyster, 2017; Wilson et al., 1994; Zrnic & Ryzhkov, 1998; among many others). However, despite over 70 years passing since the first description of animals on radar (Crawford, 1949; Lack & Varley, 1945), the widespread application of WSRs for routine monitoring of volant animals is still hampered by two key problems: (i) the useful identification or categorization of taxa (the ‘classification problem’), and (ii) the quantification of biomass and biodiversity (the ‘quantification problem’).

These twin problems have been solved—to an extent—using small biological radars (e.g. vertical looking radars, VLRs), in the UK, and insect monitoring radars in Australia), but these small radars are few and limited in their spatial coverage. VLRs are typically well‐calibrated, operate on their own, produce relatively small volumes of data that can be expertly examined, and the analysis of their observations benefits from *a priori* information about the organisms being observed. These are all factors that disadvantage the use of WSR observations as these data will come from radars whose calibrations [especially when it comes from power‐based observations such as reflectivity factor (*Z*)] will fluctuate through time and may not be consistent with other radars within the WSR network. Furthermore, the ability to make assumptions about the scatterers within the observed volumes of data and expertly examine the data is limited due to the size and diversity of the data collected across a network. Dual‐polarization Doppler WSRs have yielded promising results that enhance our ability to extract information about the size and shape of organisms, and, thus, a more accurate assessment of biodiversity that might resolve these challenges (Gauthreaux & Diehl, 2020). For these reasons, here we aim to demonstrate a novel method for analysing WSR observations that moves away from traditional, reflectivity‐based metrics to create a method that may be applied at large‐scale to benefit macro‐ecological analyses.

Meteorological monitoring has significantly improved through advances in polarimetric radar technology and an increasing understanding of the electromagnetic properties of hydrometeors (e.g. rain, hail, snow), facilitating the development of classification algorithms for those objects (Gourley et al., 2007; Lim et al., 2005; Liu & Chandrasekar, 2000; Marzano et al., 2007; Park et al., 2009; Snyder et al., 2010; Straka et al., 2000; Vivekanandan et al., 1999; Zrnic et al., 2001). Correspondingly, radar entomology, which is part of the broader discipline of aeroecology (Chilson, Bridge, et al., 2012, Chilson, Frick, et al., 2012; Vaughn, 1985), is entering a new phase, with the ability to monitor flying insects country‐ and continent‐wide using networks of dual‐polarization Doppler weather radars (Boulanger et al., 2017; Dokter et al., 2011; Gourley et al., 2007; Melnikov et al., 2015; Mueller & Larkin, 1985; Stepanian et al., 2016, 2020). This approach has proved especially valuable for observing migratory pests (Westbrook et al., 2014; Westbrook & Eyster, 2017).

Among the parameters of the observed polarimetric radar returns, the co‐polar correlation coefficient (*ρ*_HV_, an indicator of the diversity of the observed scatterers) has been found to be useful for separating bioscatterers from hydrometeors (Huuskonen et al., 2014; Zrnic & Ryzhkov, 1998). It has been shown that birds and insects may be distinguished from each other by their Doppler velocity (Huuskonen et al., 2014; Rennie et al., 2010; Zhang et al., 2005) and differential reflectivity (*Z*_DR_, an indicator of shape) (Browning et al. 2011; Melnikov et al., 2015; Mueller & Larkin, 1985; Wilson et al., 1994). Other useful quantities for bioscatterer classification include: differential phase shift (*Φ*_DP_), which is sensitive to size and shape (note that *Φ*_DP_ comprises a propagation term that dominates in rain and a backscattered component, *δ*, that dominates in bioscatterers) (Melnikov et al., 2015; Zrnic & Ryzhkov, 1998); the total measured phase difference between horizontal and vertical polarizations (*ψ*_DP_) as in Stepanian et al. (2016); linear depolarization ratio (an indicator of shape) and differential Doppler velocity (i.e. the difference in the velocity observed at the horizontal and vertical polarizations), which has been shown to differentiate effectively between hydrometeors and specific groups of birds and insects (Melnikov et al., 2014). For a full review of the use of dual‐polarization Doppler WSRs within aeroecology please see Drake and Reynolds (2012), Stepanian et al. (2016) and Chilson et al. (2017).

Novel analytical tools are needed to process the considerable volume of data from dual‐polarization Doppler WSR networks. Machine learning (ML) tools offer an opportunity to extract new, biologically meaningful information from the data but have only been applied to specific biological phenomena (Chilson et al., 2019; Gauthreaux & Diehl, 2020; Lin et al., 2019). More developed methods for species identification and application to non‐avian bioscatterers have been reported for vertical‐looking radar (e.g. Hao et al., 2020; Hu et al., 2016), but not applied to horizontally looking WSRs. Thus, new approaches are required that can classify WSR data beyond simple biomass estimates of larger organisms (Chilson et al., 2019).

Here, we demonstrate the use of a novel method (Lukach et al., 2021) applied to quasi‐vertical profiles (QVPs; Kumjian & Ryzhkov, 2012; Ryzhkov et al., 2016) of observations made by a mobile dual‐polarization X‐band Doppler WSR (Neely III et al., 2018) over an agricultural landscape in the southern United Kingdom. QVPs make the ML classification application computationally tractable while still representing a large spatial region. The ML classification results are used in conjunction with historical data collected by the Rothamsted Insect Survey (RIS) light‐trap network (Fox et al., 2021; Woiwod & Harrington, 1994). The RIS light trap network provides near‐daily, systematic monitoring data at 80 sites throughout the United Kingdom and Ireland. These moth communities have been shown to have complex long‐term temporal dynamics (Bell et al., 2020), making this taxon a prime candidate for testing innovative biomonitoring techniques that WSR could provide. Specifically, we test the following three hypotheses: (i) meteorological and non‐meteorological signatures can be differentiated in WSR data using hierarchical clustering methods; (ii) the same method can be applied to distinguish different morphotypes between groups of nocturnal bioscatterers (classification) and (iii) that the abundance of nocturnal bioscatterers observed *via* WSR provides a proxy for entomological abundance or biomass (quantification).

## Materials and Methods

### Weather radar data selection and pre‐processing

**Figure 1:**
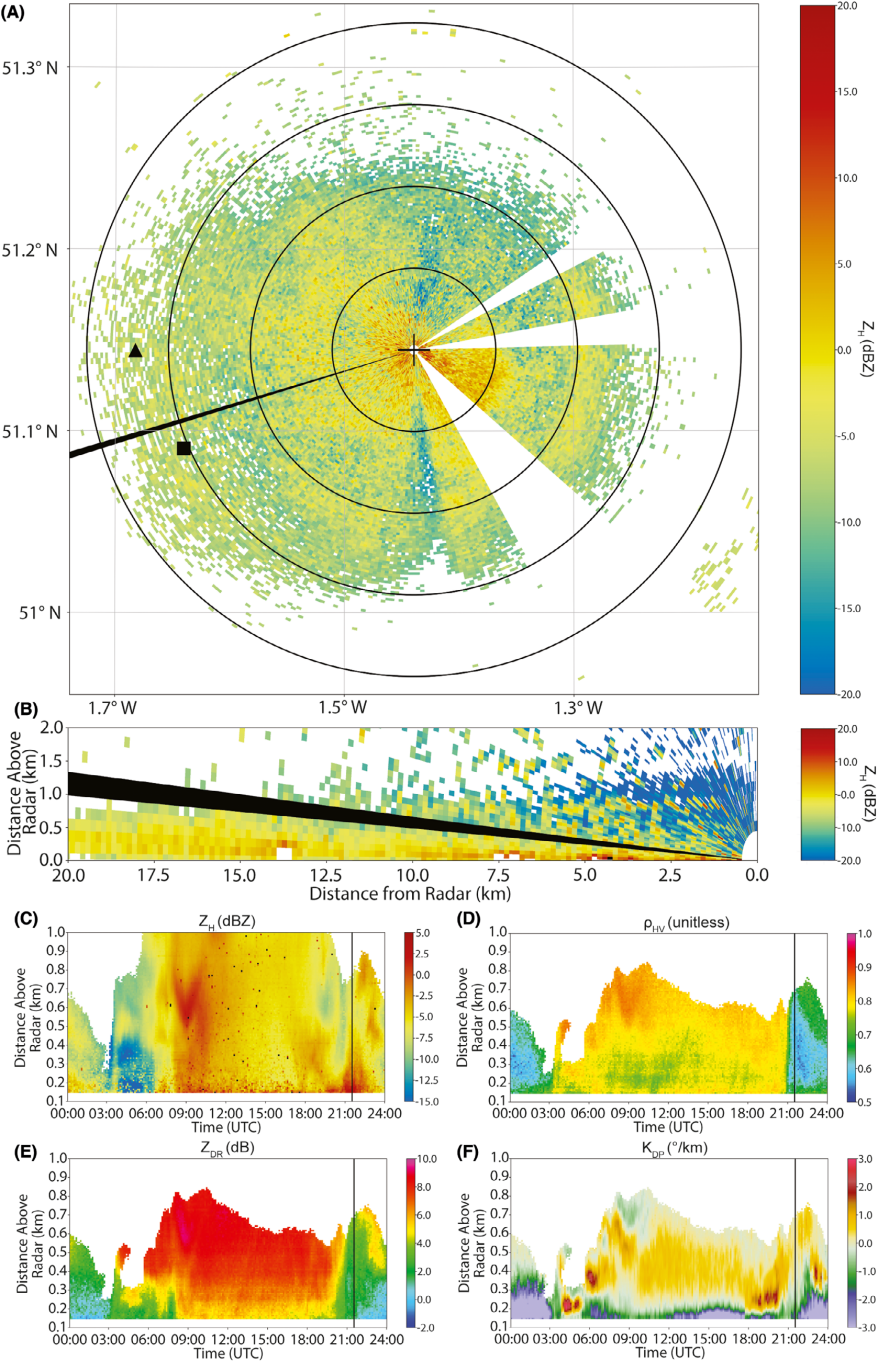
An example of typical radar data used in the analysis. Observations shown here are from June 6, 2017. (A) is a 3° elevation PPI of *Z*_H_ observed from 21:30:03 to 21:35:09 UTC. The black cross hairs indicate the location of the radar. Range rings are drawn every 5 km. The black ray indicates the azimuth of the RHI scan in (B). The black triangle is the location of the Porton Down light trap and the black square indicates the location of the Bentley Wood light trap (two sites within the Rothamsted light‐trap network). The regions where NXPol‐1 is either blocked or did not pick up any signal are white. (B) An RHI of horizontal reflectivity (*Z*_H_) taken at an azimuth of 253° observed from 21:28:29 to 21:30:03 UTC. The elevation of the PPI scan in a) and from which the QVPs are formed is indicated by the black ray. (C–F) depict QVPs of the four radar input variables used in this analysis formed from all the 3° elevation PPIs observed from 00:00:00 to 23:59:59 UTC. The approximate time of the PPI and RHI are indicated by the vertical black line. PPI, plan position indicator; QVP, quasi‐vertical profile; RHI, range height indicator. [Colour figure can be viewed at wileyonlinelibrary.com]

In this study we utilize observations from May, June and July 2017 collected by the UK's National Centre for Atmospheric Science's (NCAS) mobile X‐band dual‐polarization Doppler WSR (NXPol; Bennett, 2020). NXPol‐1 is a Meteor 50DX manufactured by Selex‐Gematronik (now Leonardo Germany GmbH), modified to operate with a larger 2.4 m diameter antenna that produces a 0.98° half‐power beam width and does not have a radome. At the time, NXPol‐1 was deployed at the Chilbolton Atmospheric Observatory (CAO; 51°8′40”N, 1°26′19.00”W). For more technical details on NXPol‐1 and the facility used to support the radar during the deployment, please refer to Neely III et al. (2018). Note that all NXPol‐1 analyses in this work utilize open‐source radar software (Heistermann et al., 2015). Specifically, we utilize routines from the following Python modules: Py‐ART (Helmus & Collis, 2016), wradlib (Heistermann et al., 2013) and scikit‐learn (Pedregosa et al., 2011). We also make extensive use of the Lidar Radar Open Software Environment (LROSE) Core Software (Dixon & Javornik, 2016). Figure 1 shows a typical example of individual horizontal (3° elevation plan position indicator (PPI); Fig. 1A) and vertical [range height indicator (RHI), Fig. 1B] scans in comparison to the QVPs created for the entirety of June 6, 2017. The first scanned volume in the path of a 3° elevation beam starts at 86 m above ground level 150 m from the NXPol‐1 and reaches a height of c. 1.57 km at a distance of 60 km from the radar.

**Figure 2:**
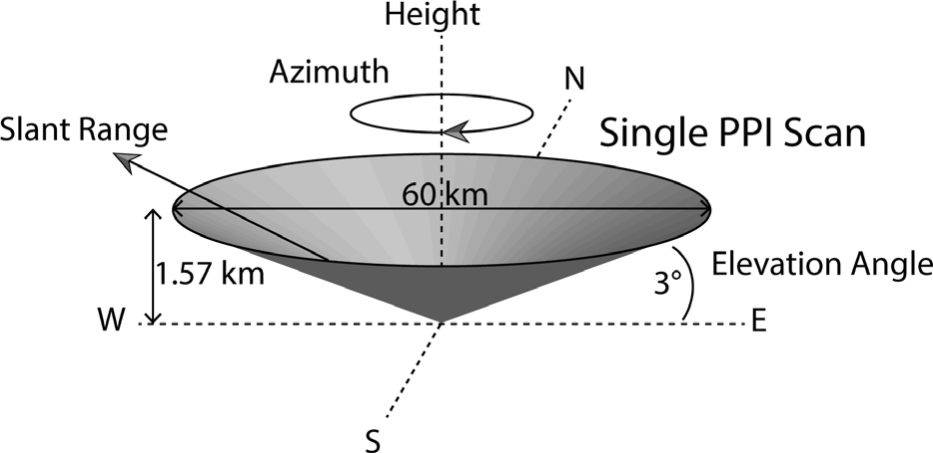
A schematic depicting the geometry of the weather radar PPI that forms the basis of a single QVP. To form a QVP, the cone of the single PPI scan is azimuthally averaged and the resulting data at each slant range is projected onto the height axis. Note that the diameter and height are given for the 3° elevation scan used in this work. This figure is adapted from similar schematics in Kumjian & Ryzhkov (2012) and Ryzhkov et al. (2016). PPI, plan position indicator; QVP, quasi‐vertical profile.

Before the cluster method is applied, the reflectivity and differential reflectivity observations are calibrated, and each PPI scan is transformed into a QVP (see Fig. 2). Individual QVPs are then concatenated together to form a dataset that represents time on the *x*‐axis and altitude on the *y*‐axis, with pixel value giving the value of the radar variable. QVPs were first used in Kumjian and Ryzhkov (2012) and Ryzhkov et al. (2016) as a way of constructing a substitute for a vertical profile from a scan conducted at constant elevation, which is a typical mode of scanning for weather radars used in operational networks.

NXPol‐1 observations from an elevation angle of 3° were used to create the QVPs in this study. The location of the NXPol‐1 at the CAO has azimuthal directions (from 45° to 185°, where 0° is North) where the radar beam is either blocked at low elevation angles due to obstacles such as other observational instruments and buildings, or where the transmitter is switched off to avoid interference (see the white or blank sectors in Fig. 1). These azimuthal directions were removed from the PPI scan data before being transformed into a QVP.

In general, utilization of QVPs has, in this context, both advantages and disadvantages. The advantages of QVPs are related to the fact that averaging values reduces statistical errors in the WSR data (Ryzhkov et al., 2016). Also, it represents well the movement of individual ‘volume targets’ when there are many individuals in the volume sampled by the WSR data at different altitudes. But this might also be a disadvantage as validation data are collected from the air column at one point of the QVP domain, and single individuals collected in this way might be not represented by ‘volume targets’ in the data. In this analysis, we chose to use the QVP representation of the data to reduce the overall dimensionality of the data. This allowed us to focus on the adaptation of the original clustering method, which is based on QVPs, to bioscatterers. Through doing this, we have ignored the azimuthal asymmetries associated with insects. This limits the analysis of the orientation of the observed organisms which would provide useful ecological information about the travel of the observed insects. For example, in directed migration with large extent a strong azimuthal variation in *Z*_DR_ will be smoothed out. Similarly, high spectrum width could indicate either multiple insect morphologies in the beam, or random flight orientation (e.g. in low wind) of similar‐sized insects. These are limitations that we must bear in mind while we analyse the resulting clusters and their characteristics. In general, the clustering method may be applied to any geometry of WSR input dataset. This includes PPIs, RHIs, volumes of PPIs and vertical columns extracted from a specific location within a PPI.

Forty 24‐h periods, belonging to seven continuous time series of data were selected for inclusion in the analysis. From these periods, 33 nights of continuous NXPol‐1 observations were selected based on the availability of light trap data (see below) and a preliminary analysis of the PPI data. The analysis allowed us to check that no bird migrations were observed over the focal nights. The nights included were: 11–14 and 31 May; 1, 14–21 and 25–27 June; and 1–11, 17–18 and 24–26 July. QVP data were only utilized for the periods of time when light trap data were also available, from civil dusk to civil dawn. QVPs of horizontal reflectivity factor *Z*_H_ [dBZ; mm^6^/m^3^], vertical reflectivity factor *Z*_V_ [dBZ; mm^6^/m^3^], differential reflectivity *Z*_DR_ [dB], co‐polar correlation coefficient Rho_HV_ [unitless] and specific differential phase *K*_DP_ [° km^−1^], were selected as input to the clustering algorithm utilized here. Example 24‐h QVP time series are shown in Figure 1.

### Bioscatterer classification algorithm

The method used in this study is based on the iterative hierarchical clustering algorithm developed by Lukach et al. (2021) for classifying hydrometeors. This algorithm (hereafter, the bioscatterer classification algorithm, or BCA) is an implementation of a ‘top‐down’ approach in which all multivariate data points are first considered as one main cluster and then split into an optimal number of sub‐clusters in a recursive procedure by applying principal component analysis (PCA) in each separate splitting step. The splitting step is performed in the ‘inner loop’, that iteratively increases the number of clusters starting from two and stops at an optimal number of clusters for a given subset of the data. The PCA reduces the dimensionality of the data and detects identifying features in the data subsets. After the features are detected, the set of clusters for the subset is passed to the ‘outer loop’ that examines the overall structure of the clusters.

The optimality of the splitting in the BCA is determined with two criteria: one ‘inner loop’ and one ‘outer loop’. The first criterion is determined as a measure of compactness based on the distances between the points and the barycenters of all clusters. The algorithm terminates the process when the clusters of the ‘inner loop’ subset reach the most compact form. These clusters are passed to the ‘outer loop’ and the posterior probability of a total clustering being true is estimated for all input points. In this way, the BCA creates a hierarchical tree that maximizes the information of the identified clusters throughout its structure. As there is no predisposition of the algorithm to focus on any particular property of the data, it is agile and is just as suited to examining bioscatterers as hydrometeors.

Advantages of this algorithm are that it is a purely data‐driven classification and that it allows for choice in the level of classification detail used in the subsequent analysis due to its hierarchical structure. The main idea behind the data‐driven clustering method is the splitting of multivariate data into an optimal number of classes according to underlying characteristics manifested in a principal components space while keeping track of the hierarchical sequence of the splitting process. Full details of the algorithm may be seen in Lukach et al. (2021).

### Entomological data selection

The output of the BCA through time was compared to observations from the RIS light‐trap network. We focused on two light trap sites located at Bentley Wood (SU253324) and Porton Down (SU223384). The distance between the two light traps is c. 6.7 km, and both are within 20 km of the CAO, providing optimal resolution within the NXPol‐1 data across both sites. For the comparison, we chose sampling dates between May and July to maximize the diversity of macro‐moth species likely to be present. Specific sampling days were chosen to exclude both significant meteorological phenomena and any moth catches involving more than one night of sampling (i.e. where the catch would be representative of two or more nights instead of a single night). This process of data selection resulted in 33 days of sampling. The Bentley Woods light trap was active across all these dates while NXPol‐1 was in operation (see above), whereas the Porton Down trap was only operational across 16 nights: 31 May; 1, 14–15, 21, and 26 June; and 3–6, 10–11, 18 and 24–26 July.

### Light trap data analysis

The two focal light traps captured 177 macro‐moth species (N = 2030 individual moths) across our 33 chosen sampling dates. Individuals are identified by trained experts who monitor the traps. We collated a morphometric trait database (Dally et al., 2021) containing mean measurements for six traits: forewing length (mm), body length (mm), thorax length (mm), abdomen length (mm), thorax width (mm) and abdomen width (mm), for each of these 177 species using digitized specimens from the collection of the Natural History Museum, London (NHM) (Natural History Museum, 2014) and the (to‐scale) colour photographic plates present in Skinner (2009) (see Supplementary Materials). We estimated a further four mean traits per species: fresh body mass (mg) (see Kinsella et al., 2020; Rydell & Lancaster, 2000), the depth of the thorax (mm) and the lateral (body length/thorax depth) and anterior (thorax width/thorax depth) aspect ratios, using several of the original mean trait values (see Supplementary Materials for a full methodology). This trait database was used to create a corresponding matrix of mean trait values per species per sampling date. Micro‐moth species were not well‐represented in the RIS light trap data, and those that were present did not have records digitized by the NHM. We therefore decided to concentrate our analysis on macro‐moth species only. As such, based on the dimensions of the sampled moths, it is highly likely that we observed a mixture of scatterers in the Rayleigh and Mie regimes, with size parameters ranging from 0.1 to 2.5. Therefore, the interpretation of the results should not be wholly considered with Rayleigh assumptions.

All entomological data analyses were carried out in R, version 4.0.2 (R Core Team, 2021). PCA, *via* the *prcomp* function, was used to reduce dimensionality within these nightly trait data. Trait data were standardized prior to the PCA, and the first two principal components (PCs) were retained. The variable loadings show PC1 as having a weak positive association with all morphometric traits, while PC2 has a strong positive association with the lateral and anterior aspect ratios (see Table S1), suggesting that PC1 indicates insect size, while PC2 indicates insect shape. Community‐weighted mean (CWM) scores for PC1 and PC2 were calculated per night, weighted by both macro‐moth abundance and biomass. Macro‐moth abundance and biomass were also summed per night. To attempt to generate a biomass variable that scales more linearly with horizontal reflectivity factor, we calculated a further variable: (mass^2^) × abundance (hereafter termed ‘mass‐squared abundance’: ‘MSA’). Finally, we used these derived variables to test for relationships between the light trap samples and the BCA clusters.

### Canonical correspondence analysis

We used canonical correspondence analysis (CCA), *via* the *cca* function in the vegan package (Oksanen et al., 2020) within R, to link the relative abundances of the different BCA clusters per night to the corresponding macro‐moth community traits derived above. The CWM scores for PC1 and PC2, together with the summed values for abundance, biomass and MSA, were used to generate four CCA models to evaluate different methods of incorporating mass and abundance into the entomological data: (i) CWM scores for PC1 and 2 were weighted by macro‐moth abundance, includes summed abundance and biomass; (ii) CWM scores for PC1 and 2 were weighted by macro‐moth abundance, includes summed abundance and MSA; (iii) CWM scores for PC1 and 2 were weighted by macro‐moth biomass, includes summed abundance and biomass; (iv) CWM scores for PC1 and 2 were weighted by macro‐moth biomass, includes summed abundance and MSA. The model that explained the greatest proportion of constrained inertia was selected. Constrained inertia is the sum of the eigenvalues associated with the constrained CCA axes (representing the explanatory variables) and is presented here as a proportion of total inertia. This is comparable to the variance within the species scores explained by the constrained CCA axes and is accepted as a measure of model fit (Økland, 1999; Ter Braak, 1986). The significance of this model, its axes and terms were assessed using permutation tests *via* the *anova.cca* function in the vegan package.

### Diversity analysis

To obtain measures of taxonomic diversity, we calculated the Shannon diversity of both the macro‐moth community and the BCA cluster ‘community’ (treating each cluster as a separate ‘species’) per night using the *diversity* function in the vegan package. To obtain a measure of macro‐moth morphometric diversity, we measured the functional dispersion (Laliberté & Legendre, 2010) of all 10 macro‐moth morphometric traits per night, using the *fdisp* function in the FD package within R (Laliberté et al., 2014; Laliberté & Legendre, 2010). Morphometric trait data were transformed into a distance matrix using the Bray–Curtis dissimilarity index using the *vegdist* function in the vegan package. We used Spearman's rank correlation to quantify relationships among these three measures of diversity, using the *rcorr* function in the Hmisc package (Harrell Jr., 2021) within R.

### Macro‐moth abundance correlations

Finally, we tested for correlations between macro‐moth abundance per night and the nightly abundance of each individual BCA cluster, as well as the total abundance across all BCA clusters per night (calculated by adding together the number of cells in the NXPol‐1 scan that are classified in one of the four bioscatterer classes), using Pearson's correlation or Spearman's rank correlation depending on whether the data conformed to the assumptions of parametric tests.

## Results

### Distinguishing between meteorological *vs.* non‐meteorological WSR signatures

**Figure 3:**
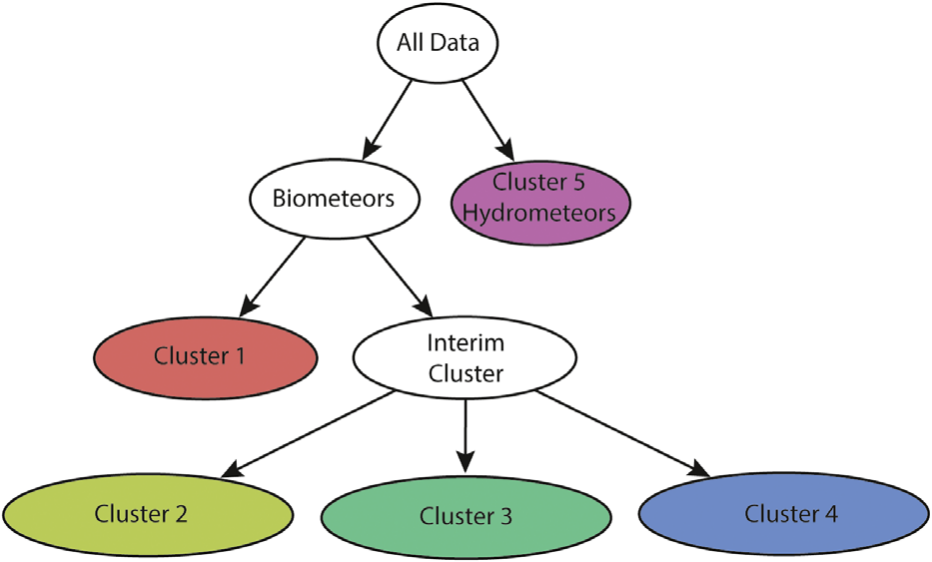
Classification tree created by the application of the BCA to the 33 nights of NXPol‐1 observations. The colour of the tree label of the clusters corresponds to the colours used in the following figures. BCA, bioscatterer classification algorithm. [Colour figure can be viewed at wileyonlinelibrary.com]

**Figure 4:**
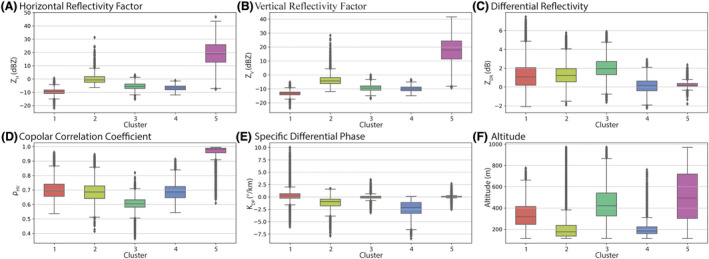
Box plots describing radar variable characteristics in (A‐E) of the five BCA clusters shown in Figure 3. A summary of the altitude at which each of the clusters is found is also included in (F). The box shows the quartiles of the dataset while the whiskers extend to show the rest of the distribution, except for points that are determined to be ‘outliers’ using a method that is a function of the inter‐quartile range. BCA, bioscatterer classification algorithm. [Colour figure can be viewed at wileyonlinelibrary.com]

The hierarchical clustering tree resulting from the BCA is shown in Figure 3, illustrating how the data were divided into five clusters. Figure 4 illustrates the mean and variability of the five radar variables used to determine each of the clusters. Figure 4 also provides a summary of the altitudes (Fig. 4F) at which each of the clusters could be found throughout the dataset to help describe the clusters further, but altitude was not included in the BCA to determine the clusters.

Note that the set of clusters output by the BCA depends on (i) the selection of the set of input variables utilized, and (ii) the set of points in the multivariate space of selected input variables. For the BCA to be stable in this context, the use of the same input data will always lead to the same output set of clusters. As in Lukach et al. (2021) we tested the BCA for stability by clustering the data multiple times and found the results to be the same.

The BCA makes a clear distinction in Cluster 5 from the remaining four clusters at the first iteration level, and the characteristics (Fig. 4) of Cluster 5 strongly suggest that this cluster represents meteorological phenomena, dominated by rain. Cluster 5 has the highest (above 0.96) Rho_HV_ mean value of all the clusters. Cluster 5 also has a narrow distribution of *Z*_DR_ values that is centred close to 0 dB. This implies that the observed radar backscatter possibly originates from spherical targets. The mean values in the horizontal and vertical reflectivities (*Z*_H_ and *Z*_V_) are highest among the other clusters (18 and 19 dBZ) but should be considered as low for meteorological observations and in most cases would represent light rain in the weather radar observations. Combining all these components together, we consider Cluster 5 as representing light rain.

As the meteorological and the non‐meteorological cluster branches were separated from each other at the first level of the BCA's tree it is easy to generalize the characteristics of all the other clusters together. The characteristics of the non‐meteorological, or bioscatterer, branch formed by Cluster 1, 2, 3 and 4 can be described by low mean *Z*_H_ and *Z*_V_ values (between 0 and −12 dBZ), slightly positive mean *Z*_DR_ values (between 1 and 2 dB) and low Rho_HV_ mean values (<0.7).

From the bioscatterer clusters, Cluster 1 has the lowest mean reflectivity values (−10 dBZ for *Z*_H_ and −12 dBZ for *Z*_V_) and a mean *Z*_DR_ value near 1 dB. These characteristic values suggest that data points belonging to Cluster 1 should come from observations comprised of ensembles of bioscatterers with low number densities (i.e. few animals in the observed radar volume) that have relatively small bodies that are slightly elongated and horizontally oriented. Conversely, Cluster 2 has the highest mean reflectivity values among all the bioscatterer clusters (close to 0 dBZ in both *Z*_H_ and *Z*_V_). Like Cluster 1, Cluster 2 has a mean *Z*_DR_ value of ~1 dB and a mean Rho_HV_ value of ~0.7, but Cluster 2 is also distinguished by having a negative mean *K*_DP_ value. This suggests Cluster 2 is comprised of observations of relatively dense ensembles of larger, horizontally oriented bioscatterers with slightly elongated bodies. The inferred larger size of the bioscatterers comprising this cluster is also supported by the fact that Cluster 2 is found closer to the surface than Cluster 1 (Helms et al., 2016; Hespenheide, 1975, 1977; Smith et al., 2000).

The mean reflectivity values of Clusters 3 and 4 are intermediate to those of Clusters 1 and 2. Thus, Clusters 3 and 4 are mostly separated from the other clusters by the remaining polarimetric radar variables. In particular, Cluster 3 has the lowest mean Rho_HV_ value (~0.6) and the highest mean *Z*_DR_ value (~2 dB). Combined with its other characteristics, these values suggest that Cluster 3 represents observations of bioscatterers with low number density that have elongated body shapes that are highly uncorrelated in the observed air volume. The uncorrelated signature of Cluster 3 may be partially due to variations in body geometry caused by wing beating which could influence the bioscatterers backscattering characteristics. Unlike the other three bioscatterer clusters, Cluster 4 has a near 0 dB mean *Z*_DR_ value. Thus, combined with also having the most negative mean *K*_DP_ value (~ −2.5°km^−1^), Cluster 4 likely comprised a collection of bioscatterers with low numbers of larger but more spherical bodies.

**Figure 5:**
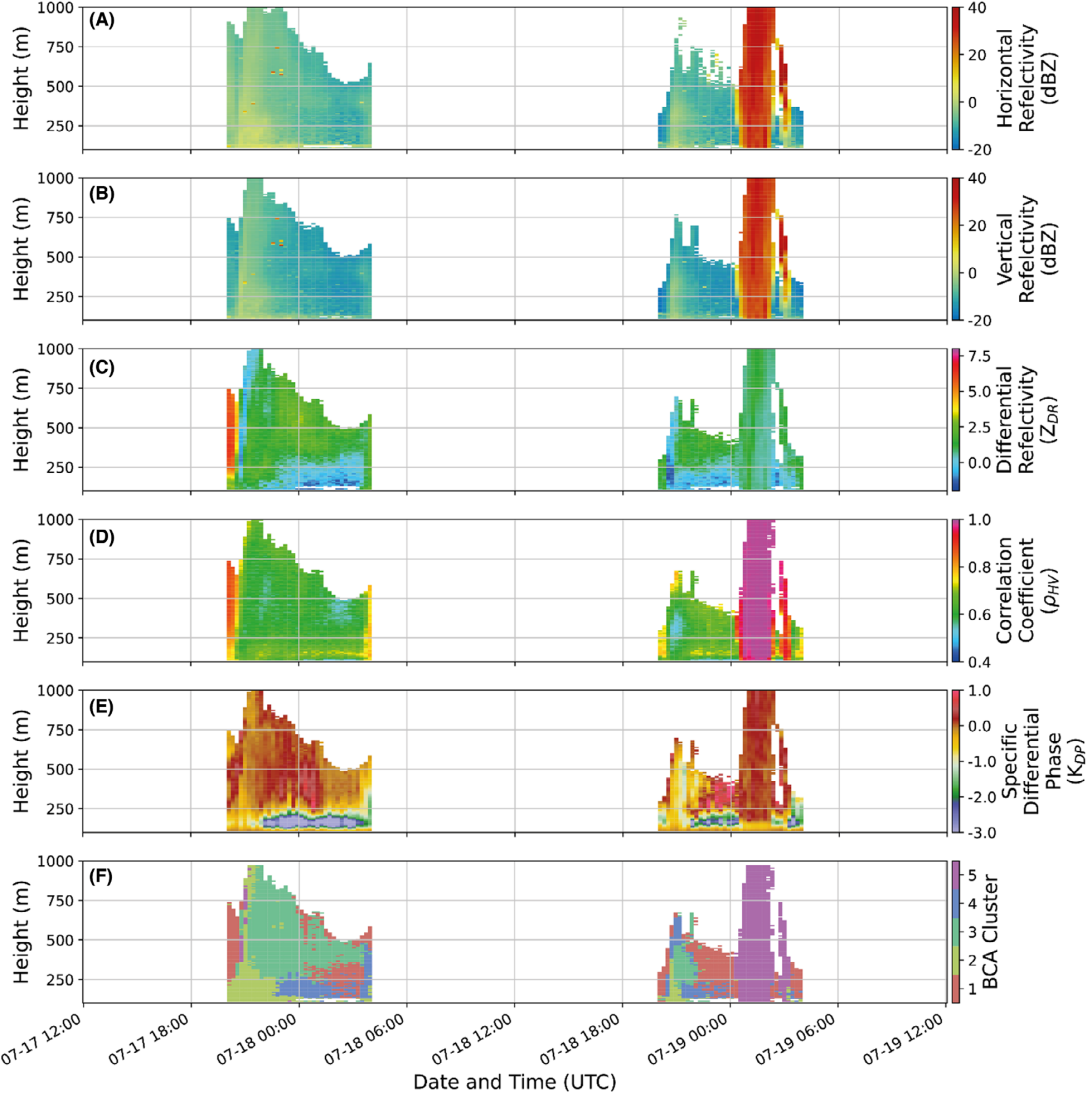
Time series of the input data (A) horizontal reflectivity, (B), vertical reflectivity, (C) differential reflectivity, (D) correlation coefficient, (E) specific differential phase) and the resulting distribution of the 5 BCA clusters (F) across 2 nights of QVP observations. BCA, bioscatterer classification algorithm; QVP, quasi‐vertical profile. [Colour figure can be viewed at wileyonlinelibrary.com]

If we assume that the clusters are primarily representative of insects, we can think of Cluster 1 as representing smaller individuals that are homogenous in terms of their morphology, with slightly longer, slimmer body shapes—potentially reminiscent of smaller Diptera. This cluster is ubiquitous within the air column, being present between ~100 and 800 m in altitude (Fig. 5), but is present at low densities. Cluster 2 represents insects that are similar in body shape to those in Cluster 1 but are larger and are present at greater densities. This cluster is similarly present throughout the air column. Cluster 3 represents insects that are very elongate in shape but are otherwise more diverse in morphology than Clusters 1 and 2, indicating a mixture of taxonomic groups present predominately between 300 and 500 m and at lower densities. Whereas Cluster 4 represents insects that are morphologically homogenous, but with larger, more spherical bodies. This cluster is present at lower densities and is found closer to ground‐level (<300 m).

The ecological interpretation of these clusters in the context of ‘biodiversity’ is not straightforward. An increase in cluster 3 at the expense of clusters 1 and 2 may involve an increase in overall diversity as one of the features of cluster 3 is that the constituent objects are diverse in size and shape. However, an increase in the diversity of clusters will still tend towards an increase in the broader diversity of insect morphotypes, as the clusters each represent different community structures drawn from the wider species pool. Further work will be needed to explore the extent to which species overlap in their memberships of different clusters and cluster‐specific spatio‐temporal distributions.

Linking these clusters to specific (groups of) taxa will require a series of cross‐validation studies. These studies could involve VLR or other entomological radars that can identify species at different heights. However, those VLRs suffer from similar issues to the WSRs in that they can vary in calibration and power. Aerial netting can be used to capture animals in the air column, but this is a considerable logistical exercise that captures relatively few animals. Finally, it is possible to use natural experiments during mass emergences or migrations of known insect taxa to explore the link between radar measurements and those specific morphotypes. Examples of such natural experiments include aquatic insect emergence (e.g. Stepanian et al., 2020) or ant nuptial flights.

The main difficulty in direct attribution of the detected clusters to possible classes based only on the mean characteristics of the clusters, is related to the nature of WSR observations. These are influenced by the size, shape, number density and dielectric properties of the targets as well as the wavelength of the radiation being utilized by the WSR. This multitude of influences makes any property‐based attribution only tentative, and a proper assignment requires support of a detailed analysis and validation based on ground‐based or aerial observations of the morphology of nocturnal insect taxa.

Figure 5 shows the results of the BCA applied to two consecutive nights of NXPol‐1 observations from the 33 days used in this study. In addition to the clusters, we also show the time series of the input data to the BCA to enable a comparison of the observed variables to the output of our algorithm. These nights were chosen as a representative sample of the entire time series. The vertical and temporal pattern of the clusters on the first night (July 18, 2017) is typical for nights of high general bioscatterer activity and no significant meteorological phenomena. Generally, Cluster 1 is always detected first (and on nights with little activity may be the only cluster observed) and rises from the near surface before sunset (cut off here and not included in the analysis) then moves to higher altitudes later in the night. This is followed by the dominance of Cluster 2 near the surface and Cluster 3 at higher altitudes. Cluster 4 typically always appears after Cluster 3 near the surface. Cluster 1 also generally makes a reappearance before dawn after the altitudinal extent of Cluster 3 has decreased. The second night (July 19, 2017) shows a similar pattern, but in this case the time series is interrupted by a brief period after midnight where Cluster 5 dominates. Comparison to observations from the UK Met Office's WSR network confirm that widespread rainfall moved from the south of the UK northwards during this period.

### Bioscatterer classification and abundance

The BCA results in Figure 3 support the existence of four discrete, radar‐derived bioscatterer clusters that vary in their observed scattering characteristics which is assumed to be caused by differing morphometric characteristics of the observed volumes of organisms; Figure 4. The four BCA clusters were tested for their relationships with the light trap samples using three complementary approaches. First, for each night of sampling, macro‐moth community traits were compared against the relative frequencies of the BCA clusters using CCA. This test allows us to establish whether it is possible to explain statistically the variation in the relative abundance of BCA clusters using known traits data in the local macro‐moth community, based upon similarity of morphology, abundance and biomass.

**Figure 6:**
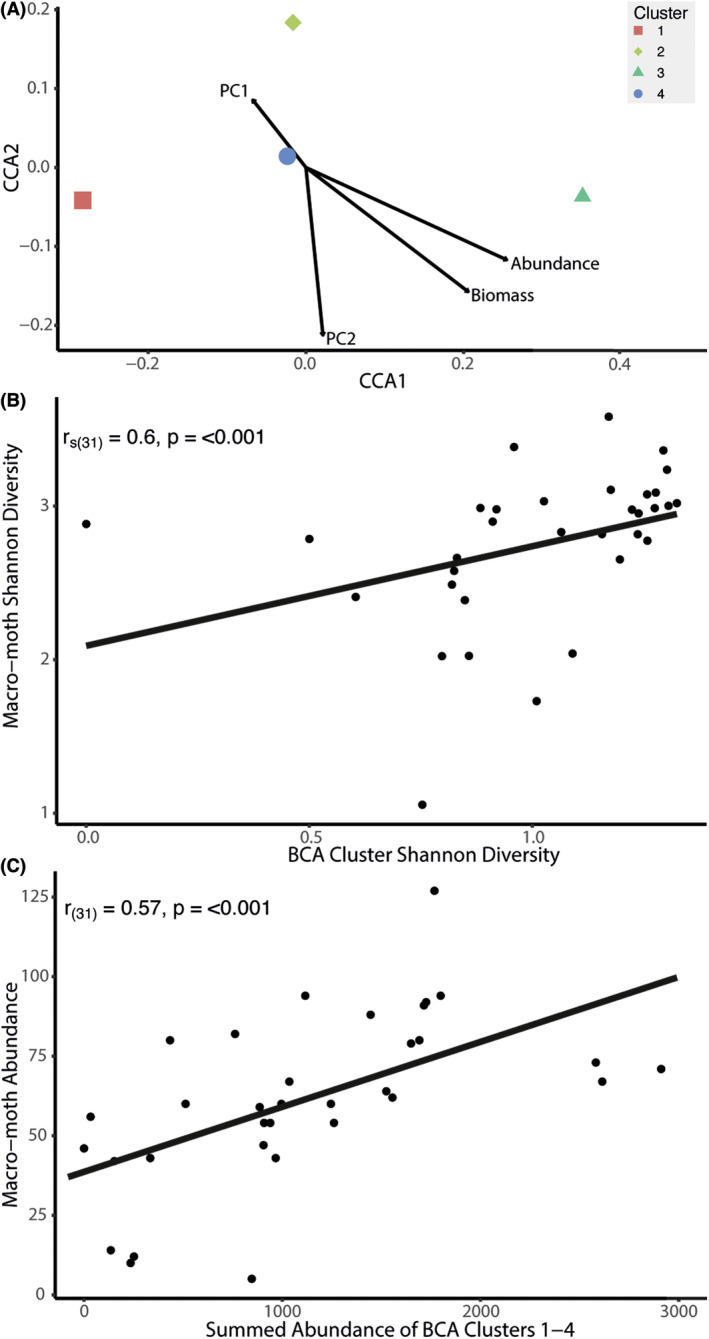
(A) CCA biplot showing the four BCA clusters and their relationship to four derived macro‐moth community traits: community‐weighted mean values for PC1 and PC2 (weighted by biomass) per night, and summed abundance and biomass per night, indicated by the four labelled arrows; (B) scatterplot showing the relationship between the taxonomic (Shannon) diversity of both the macro‐moth community and the BCA cluster community present per night over our two focal RIS light‐trap sites; (C) scatterplot showing the relationship between summed macro‐moth abundance per night and the summed abundance of bioscatterers described by BCA clusters 1–4 (calculated by adding together the number of cells that are classified as one of the four bioscatterer classes) per night over our two focal RIS light‐trap sites. BCA, bioscatterer classification algorithm; RIS, Rothamsted Insect Survey; CCA, canonical correspondence analysis. [Colour figure can be viewed at wileyonlinelibrary.com]

Of the four CCA models, model 3 (Table 1) explained the greatest proportion of constrained inertia (0.251) and provided a statistically significant prediction of the BCA clusters based on community traits [*F*_(4,28)_ = 2.339, *P* = 0.043]. Approximately 89% of the constrained inertia of this model was explained by the first axis, although this axis did not statistically predict BCA clusters alone [*F*_(1,29)_ = 8.647, *P* = 0.064]. This axis was characterized by moderate‐to‐high scores for summed abundance (eigenvalue weighting: 0.88) and summed biomass (0.71) per night. BCA Cluster 3 displayed a weak positive score for this axis (0.35) (Fig. 6A). Permutation tests show that summed macro‐moth abundance was the only significant predictor of BCA clusters in the model [*F*_(1,28)_ = 6.511, *P* = 0.015] when all terms were tested sequentially, but that no terms were significant when each was tested independently. Overall, this indicates that, while BCA cluster 3 can be typified by an increased abundance in comparison to the other clusters, we can make no assumptions regarding the morphology of the bioscatterers characterized by the BCA clusters.

**Table 1:** The proportion of constrained inertia explained by each of the four CCA models, together with corresponding pseudo‐*F* statistic, degrees of freedom and *P*‐value. CCA, canonical correspondence analysis.

Model	Proportion of constrained inertia explained	Pseudo‐*F* statistic	df	*P*
[1] CWM PC1 & PC2 (abundance); summed abundance & biomass	0.224	2.023	4, 28	0.082
[2] CWM PC1 & PC2 (abundance); summed abundance & MSA	0.202	1.766	4, 28	0.131
[3] CWM PC1 & PC2 (biomass); summed abundance & biomass	0.251	2.339	4, 28	0.043
[4] CWM PC1 & PC2 (biomass); summed abundance & MSA	0.244	2.259	4, 28	0.062

CWM, community‐weighted mean; PC1 and PC2, the scores for the first and second principal components from the PCA.

The second approach compared the diversity of BCA clusters with the diversity of macro‐moths captured per night. Diversity was expressed both as taxonomic diversity (Shannon diversity, based on relative frequencies of different macro‐moth species or radar clusters) and functional dispersion (a measure of macro‐moth morphological variability). Results showed a strong positive correlation between the taxonomic diversity of both the aerial macro‐moth community and the BCA clusters per night [*R*_s(31)_ = 0.60, *P* < 0.001] (Fig. 6B).

Finally, correlation tests showed significant positive relationships between macro‐moth abundance and the abundance of BCA clusters 2 (*R*_s(31)_ = 0.5, *P* = 0.003), 3 (*R*_s(31)_ = 0.73, *P* < 0.001) and 4 (*R*_s(31)_ = 0.53, *P* = 0.002), as well as total abundance across clusters 1–4 (*R*_s(31)_ = 0.57, *P* < 0.001), per night (Fig. 6C).

## Discussion

While the potential for the use of dual‐polarization Doppler WSRs has been recognized for the study of aerial insects (Bauer et al., 2019; Didham et al., 2020), our study represents the first demonstration that classification algorithms can extract meaningful community data from WSR data without *a priori* information. Our results show that meteorological scatterers can be distinguished meaningfully from biological scatterers using an unsupervised algorithm if both types of scatterers are represented in the original data. We go on to demonstrate the important next step: that biological information from WSRs has ecological meaning in terms of the abundance and diversity of insects at or near ground level. While we do not argue that the bioscatterers in the radar data correspond directly with the community of moths at ground level, we have provided strong evidence for a correlation between the two communities that is likely caused by one or more shared drivers (e.g. weather, phenology, etc.). As such, our findings suggest that the radar‐derived measure of abundance and diversity of nocturnal insects can be used to predict—if not measure—ground‐level biodiversity.

A range of algorithms has been proposed to extract data on bird movements from WSRs. The vol2bird algorithm works based upon the velocities of birds relative to hydrometeors using volume velocity profiling (Dokter et al., 2011); though it is recognized that this would not be as useful for insects as many species move passively with the wind. Meanwhile other techniques have relied on convolutional neural networks to process spatiotemporal arrays of radar data (Lin et al., 2019). In comparison to the wider literature, the BCA used here is capable of distinguishing hydrological from biological scatterers at least as well as existing supervised classification methods but, importantly, without any additional assumptions regarding how these classes are represented in the original data. The BCA here needs no training data to inform the clustering and can be applied to all QVP‐data universally—requiring no adjustments for different data types. The BCA additionally probes the data to explore the extent of morphological (and hence, potentially, taxonomic) differentiation present in the air column. However, the output of the clustering is heavily reliant upon the quality and content of the input data. If certain data classes (e.g. hydrometeors) are not present, or are present but in very low densities, then these classes have a low chance of being represented in the set of final clusters.

This well‐supported differentiation between scatterers opens the possibility for the quantification of biomass regardless of the taxonomic resolution of the data, as has been done in the case of bird migrations (Chilson et al., 2019; Lin et al., 2019). Even if our analysis had not demonstrated additional value in the data in terms of estimating biological diversity, quantification of abundance would be a useful insight for future research. The fact that we can extract relatively strong correlations between entomological measures of abundance and measures of overall radar reflectivity also raises the exciting prospect of using WSR observations as a general proxy for biomass at a large scale. Such work using WSRs has previously been focused on mass emergences (Stepanian et al., 2020) or swarming events (Westbrook et al., 2014), but, as yet, there has been no attempt at creating a regional map of standing insect biomass that is relevant for ground‐level ecosystems.

Additionally, our findings show that the taxonomic diversity of moths on the ground correlates with the diversity of the bioscatterer clusters. This suggests that the taxonomic or morphological resolution at which WSRs can operate—at least in the case of nocturnal insects at our study sites—can yield ecologically meaningful insights. However, there is morphological variation within our bioscatterer clusters that is too subtle to be linked to explicit morphological variability in the macro‐moth community data. Past work has demonstrated that it is difficult to differentiate birds and insects in WSR data (see discussion and methods in Nussbaumer et al., 2021). This is primarily driven by the lack of *in situ* observations to compare directly to WSR observations. In this case, the interpretation of the results is further complicated due to the observed bioscatterer clusters most likely being a mixture of both Rayleigh and Mie scatters, so the simple relations used in the interpretation of the polarimetric quantities that represent the characteristics of cluster may not hold. Without additional *in situ* data and electromagnetic modelling, these results will remain uncertain. Nevertheless, we still demonstrate how the algorithm may be utilized as a tool to examine such a dataset.

In this analysis, the *in situ* observations come from RIS light traps that are estimated to sample within a radius of 30–50 m and feature an opaque covering that obscures the bulb from insects flying at high altitudes (Bell et al., 2020). Since only macro‐moth species are counted and identified from among all insect taxa attracted to the traps, the light trap samples are considered to provide a standardized measure of the relative abundance of local macro‐moth populations. They are not, however, necessarily representative of broader crepuscular and nocturnal aerial insect communities, which may comprise a mixture of dipteran, coleopteran, neuropteran, trichopteran and ephemeropteran species, in addition to both macro‐ and micro‐moth species, depending on the time of year, habitat and environmental factors (Chapman et al., 2004; Wakefield et al., 2018; Wickramasinghe et al., 2004; Wood et al., 2010). Further, we would also not expect actively migrating moth species to be well‐represented within the RIS light trap data since these migrations occur at high altitudes (Wood et al., 2009). However, migratory moth species are often found within RIS light trap samples, representing individuals that are either yet to begin their migration or that finished migrating and are engaging in local flights in search of food etc.

In addition, the QVP representation of bioscatterer abundance is calculated over a much larger area (~2800 km^2^) than that estimated to be sampled by each light trap, providing a landscape scale measure of aerial bioscatterer activity. This difference in the scale of spatial sampling, together with the focus on macro‐moth species in the light trap data, may be responsible for our inability to relate moth morphometric diversity to the different BCA clusters since, depending on the level of landscape heterogeneity surrounding our two focal light traps, aerial insect (and macro‐moth) diversity may differ significantly within the area represented by the QVP. However, the RIS light‐trap network remains the only source of systematic nocturnal insect monitoring data in the United Kingdom. Bird and bat populations will also have contributed to patterns of biological activity on WSR observations (Boero et al., 2020; Nilsson et al., 2019; Stepanian et al., 2019) but, as no large‐scale bird migrations were identified in the data and no large populations of high‐flying bat species have been observed within the study area, the impact of this on our analysis is minimal (NBN Atlas, 2021).

However, one promising method may be the linking of morphology with radar scattering based on electromagnetic modelling, although this approach is in its infancy (Drake et al., 2017; Mirkovic et al., 2016, 2018). Electromagnetic modelling would allow researchers to make ‘bottom‐up’ predictions of radar echoes based on size and shape in a range of taxa, effectively starting with a search image derived from the modelling and searching through data for that specific object. Such an approach would be complementary to the ‘top‐down’ approach shown in our study of taking a series of radar observations and categorizing combinations of values. At present, in our data, while there is some evidence that the ensembles of bioscatterers represented by the clusters vary in size and shape, this variation appears to be too broad (or our taxonomic sampling too specific) to detect a correlation between radar‐observed scattering characteristics and macro‐moth morphology. This is further complicated by the need to account for Mie scattering in our interpretations.

The scattering attributes of individual radar targets, relating to their size, shape and mass, have been previously used to distinguish different insect taxa in vertical‐looking radar (VLR) scans (Chapman et al., 2002; Drake, 1984; Reynolds et al., 2005; Smith et al., 2000; Wood et al., 2006, 2009). Following the longstanding success of VLR radar analysis, dual‐polarization weather radar has been identified as a tool (Bauer et al., 2017, 2019; Chilson, Bridge, et al., 2012, Chilson, Frick, et al., 2012; Dokter et al., 2011; Gauthreaux et al., 2008; Shamoun‐Baranes et al., 2019; Stepanian et al., 2016, 2020) for examining biodiversity over larger spatial areas if the information within the backscattered electromagnetic signals could be tied to meaningful biological and ecological information. Following this path, our analysis represents the first attempt to demonstrate this link using detailed ground‐truthing data in nocturnal insect communities. Our results provide strong evidence for the value of dual‐polarization weather radar data to the monitoring of nocturnal biodiversity, although there is no reason to expect that the approach would be limited to that application.

Widespread application of polarimetric Doppler WSRs for the monitoring of standing insect biomass at an international scale and at high temporal and spatial resolutions would inform insect conservation legislation and practice at a scale that is currently unprecedented. Much of the promise of weather radars for biological monitoring can be unlocked with greater advances in algorithmic classification tools and the adoption of standardized data storage formats that facilitate wider access (Bauer et al., 2017; Gauthreaux & Diehl, 2020). We note that although the WSR used in this study is an X‐band system, S‐ and C‐band are the most common frequency bands utilized WSR networks. As the insects, especially moths and other ‘macro‐insects’, are fairly large in X‐band (~3 cm wavelength) and relatively smaller targets in C‐ and S‐band (~5 and ~10 cm in wavelength), the polarimetric quantities and even reflectivity may have significant differences from one radar band to another. Even so our algorithm would still be readily applicable and should be able to easily discern between differing clusters within the observed radar volumes as it does not require *a priori* information. In fact, the algorithm would even more beneficial when trying to combine observations of a single volume from two radars of differing frequency as the framework provided by the algorithm would allow for a combined classification of the polarimetric quantities at both wavelengths. Such an approach should be cross‐validated using the same sorts of insect monitoring datasets we apply in the present study from the entirety of Rothamsted Research's light and suction trap networks from across the UK.

However, many of the most pressing issues to which WSR observations can be applied, for example the monitoring of swarms of destructive pests and the tracking of biodiversity trends in megadiverse areas, are in the Global South (Boero et al., 2020) where the infrastructure and resources for the creation and maintenance of radar networks are limited. Hence, most applications in those areas have relied upon remote sensing from satellite data (Schulte to Bühne & Pettorelli, 2018) which hitherto has shown limited abilities for monitoring aerial biodiversity. As the WSR networks are developed in those countries, the integration of aeroecological algorithms like ours will help to maximize the return on that infrastructure investment.

## Conclusion

Polarimetric Doppler weather radars have the potential to resolve long‐standing issues in ecological monitoring. We provide an attempt to classify aerial nocturnal insects (bioscatterers) using X‐band dual‐polarization weather radar observations with ground truthing using high‐resolution ecological community data. We demonstrate that our BCA, based on a novel iterative hierarchical clustering technique, easily distinguishes meteorological from non‐meteorological phenomena. Additionally, the clusters corresponding to bioscatterers were primarily separated along axes of biomass and aspect ratio, although strong support only exists for only a subset of the clusters. This indicates a relatively weak ability (in this analysis) to provide higher taxonomic resolution for these bioscatterers. This result is most likely due to the strong influence of Mie scatterers and the lack of *in situ* data within the observed radar volume (i.e. above the ground). Finally, and most interestingly, we show that the diversity of radar‐observed bioscatterer clusters correlates significantly and positively with the diversity of moths that dominate the nocturnal air column. Taken together, these results demonstrate a proof of concept for the application of dual‐polarization WSR networks to monitor UK insect communities at a national scale, with the potential for dissemination wherever there are comparable operational WSR networks. These novel data sources may represent a step‐change in the monitoring of insect populations by providing a much‐needed international, standardized source of information to identify and diagnose changing population trends.

## Supporting information


**Data S1.** Supplementary methods, including definitions of radar variables and details of moth morphometric measurements.

**Table S2.** The forewing length (mm), thorax width (mm) and thorax depth (mm) of 37 macro‐moth species (65 individual macro‐moths).
