# Supplementary material for: The development of an unsupervised hierarchical clustering analysis of dual‐polarization weather surveillance radar observations to assess nocturnal insect abundance and diversity
Source: Remote Sens Ecol Conserv. 2022 May 24;8(5):698–716. doi: 10.1002/rse2.270 (PMC9790603; doi:10.1002/rse2.270)
Supplement: Supplementary file 1 — Data S1. Supplementary methods, including definitions of radar variables and details of moth morphometric measurements. [file RSE2-8-698-s002.docx]

**Supplementary Information:**

**A Description of the Dual-polarisation Doppler Weather Radar Variables Used in the BCA**

The five weather radar variables used as input to the BCA include:

- Horizontal reflectivity factor Z_H_ [dBZ], which is proportional to the sum of the sixth power of the dimension of the target parallel to the ground in a unit volume of space (Figure 1c).
- Vertical reflectivity factor Z_V_ [dBZ], which represents the same as Z_H_ but observed in the vertical direction. These two variables will both have higher values if the number and the size of the observed targets is larger, and where the wavelengths are much larger than the size of the scatterer. The composition of the target, influencing its electromagnetic properties, also plays a role.
- Differential reflectivity Z_DR_ [dB], which is the ratio of the reflectivity observed with transmitted and received signals of horizontal polarisation to that observed with signals of vertical polarisation. In combination with Z_H_ and Z_V_, for wavelengths much larger than the size of the scatterer, it provides an estimate of the physical shape of observed targets. More spherical targets, like small rain drops, would provide a Z_DR_ value of close to 0 dB, but most probably would also have low reflectivity values. Targets with more elongated shapes, oriented horizontally, would provide positive values of Z_DR,_ while a vertical orientation of the same shapes would provide negative values of Z_DR_.
- Co-polar correlation coefficient ⍴_HV_ [unitless], defines the relationship between the magnitude and phase of the measurements of the horizontal and vertical channels. ⍴_HV_ would have a value of 1 for stationary scatterers contained in a sampled volume. If the shape, orientation, or motion of the scatterers becomes more variable at each polarisation (horizontal and vertical) the signals will become less correlated and ⍴_HV_ will decrease. The same effect can be observed with heterogeneously dispersed targets inside the sampled volume (non-uniform beam filling effect). As the shape, orientation, body position and spatial distribution of biological targets are highly variable, they should be represented by lower ⍴_HV_ values.
- Specific differential phase K_DP_ [° km^−1^], which is defined as a change or delay between horizontal and vertical phase-shift with respect to the change in range - the K_DP_ values are highest where the phase shift occurs. Positive K_DP_ values indicate a greater phase shift in horizontal than vertical signals, which would mean more horizontally oriented targets of larger sizes in the scanning volume.

A full review of the concepts of dual-polarisation Doppler weather radar applied to ecological monitoring may be found in Stepanian et al. (2016).

***The Macro-moth Morphometric Trait Database***

Our primary data consists of macro-moth records from two Rothamsted Insect Survey (RIS) light traps, located at Bentley Wood (Lat: 51.090, Long: -1.640) and Porton Down (Lat: 51.144, Long: -1.682) (listed as Bentley Woods and Porton Down III by the RIS). Across our 33 chosen sampling dates in 2017 (11-14 and 31 May; 1, 14-21 and 25-27 June; and 1-11, 17-18, and 24-26 July), 177 macro-moth species were sampled (N=2030 individual moths).

Since the RIS light-trap network does not retain and store physical samples, our morphometric trait database was primarily constructed using images of these 177 macro-moth species, namely digitised specimens from the collection of the NHM (Natural History Museum, 2014), and the (to-scale) colour plates from *Colour Identification Guide to Moths of the British Isles* (Skinner, 2009).

We chose to measure six morphometric traits: forewing length, body length, thorax length, abdomen length, thorax width, and abdomen width (see Supplementary Figure 1); all of which were measured in millimetres. These traits were chosen to best characterise the overall size and shape of a moth’s body and were based upon the traits used to estimate lepidopteran body mass in García-Barros (2015).


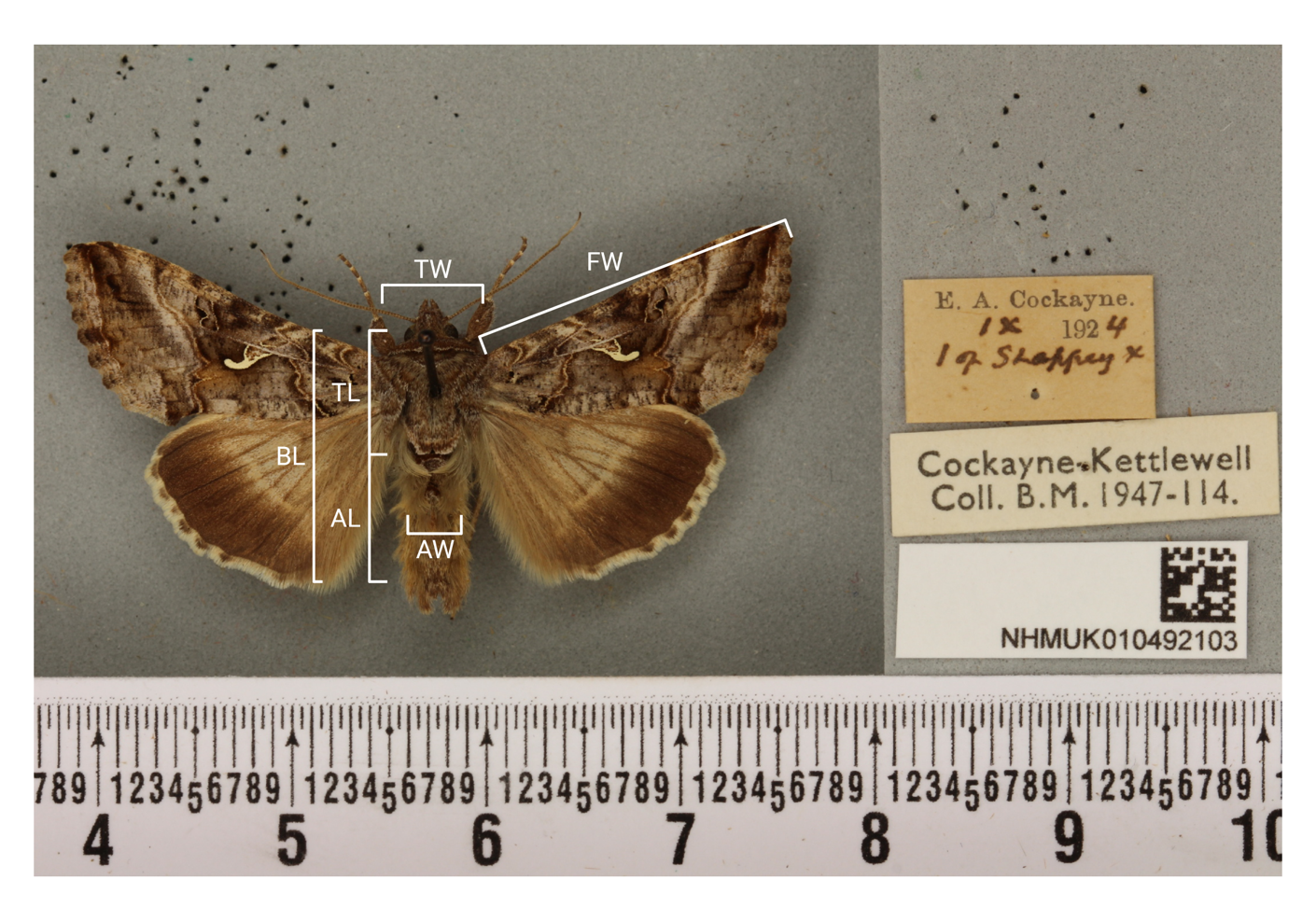


**Supplementary Figure 1.** An illustration of the morphometric trait measurements taken from each macro-moth specimen: forewing length (FW), body length (BL), thorax length (TL), abdomen length (AL), thorax width (TW), and abdomen width (AW), created with BioRender.com. The image represented is of the Silver Y, *Autographa gamma* (Linnaeus, 1758) (Natural History Museum, 2018).

For each macro-moth species within our primary dataset that had specimens with images digitised by the NHM, we randomly selected five specimens for measurement. Where a species displayed sexual dimorphism that resulted in significant differences in overall body size and/or shape between sexes, we randomly selected five specimens per sex. Sexual dimorphism was identified via a range of sources, including Skinner (2009) and UKMoths (ukmoths.org.uk). Trait measurements were taken using ImageJ software (Schneider et al. 2012). All digitised images from the NHM include a scale bar that allowed us to calibrate our measurements using ImageJ.

Where a species within our primary data had no specimens with images digitised by the NHM, we took measurements from the (to-scale) colour plates present in Skinner (2009). Again, five images were randomly selected per species for measurement, except where significant sexual dimorphism was present within a species, in which case five individuals per sex were randomly selected. Skinner (2009) often included fewer than five images per species (or per sex per species) and therefore, in many cases, we simply measured as many images per species as we could, inclusive of both sexes. Measurements from Skinner (2009) were made with Duratool digital callipers (resolution: 0.1mm; accuracy ±0.2mm).

Four macro-moth species displayed species dimorphism but had no identifying information regarding the sex of the randomly selected specimens within the NHM digitised collection. For these four species (*Alcis repandata*, *Hepialus lupulinus*, *Lymantria monacha*, and *Photedes minima*), additional measurements were taken from Skinner (2009) for five male and five female specimens (or as many specimens per sex per species as were present).

In addition to the six morphometric traits, the sex of each specimen measured was recorded (if known), as well as a unique identifier for the image (a permanent URL for each digitised NHM record, or the plate and specimen number for records taken from Skinner, 2009). Due to human error, fifteen of our specimens do not have a unique identifier associated with their records and are instead marked as “NA”.

Three species aggregates were also present in the species counts within the RIS light trap data (in addition to the 177 species): *Eupithecia* species, *Oligia* species, and “unidentified macro spp”. In each case, we took the mean average of each morphometric trait across all measured individuals within each genus (*Eupithecia* and *Oligia*), or across the whole dataset (“unidentified macro spp”), and used these as proxy trait measurements for these records.

We used these six trait values to estimate a further four morphometric traits per individual: fresh body mass (mg), thorax depth (mm), and the lateral and anterior aspect ratios.

*Body mass (mg)*: Following Kinsella et al. (2020), we used forewing length to estimate the dry body mass (mg) of each macro-moth individual within the morphometric trait database. Kinsella et al. (2020) found that forewing length was a strong predictor of dry mass in macro-moths and provide family-specific model parameters (slope and intercept estimate values) for the following families: Crambidae, Erebidae, Geometridae, and Noctuidae, as well as an “other families” aggregate grouping. These can be entered into the following formula to estimate dry body mass:

*ln(body mass) = (ln(forewing length) × (“other families” slope + family slope adjustment)) + (“other families” intercept + family intercept adjustment)*

Further details of this equation and the specific parameters involved can be found in Table 1 of Kinsella et al. (2020). An exponential was applied to the final value to remove the natural logarithm. Fresh body mass was estimated for each moth specimen using the 1:0.36 conversion ratio between fresh and dry mass for “macrolepidoptera” in Rydell and Lancaster (2000).

*Thorax depth (mm)*: All the images from which our trait measurements were taken presented a dorsal perspective of each moth, leaving us with no concept of body “depth”. We chose to estimate thoracic depth, the distance between the dorsal and ventral thoracic surfaces, since the thorax is the body segment least prone to deformation as an insect specimen dries.

To estimate this trait, we collated measurements of the forewing length, thorax width, and thorax depth of 65 pinned macro-moth specimens (n), encompassing 37 species (N) from the three predominant macro-moth families present in our data set: the Noctuidae (N=13, n=24), Geometridae (N=14, n=25), and Erebidae (N=10, n=16). This additional trait data is included in Supplementary Table 2.

**Supplementary Table 2**. The forewing length (mm), thorax width (mm), and thorax depth (mm) of 37 macro-moth species (65 individual macro-moths). Morphometric trait measurements were taken from pinned specimens using Duratool digital callipers (resolution: 0.1mm; accuracy ±0.2mm) by Dr Charles Fletcher from his extensive macro-moth reference collection.

| Family | Species | Forewing length (mm) | Thorax width (mm) | Thorax depth (mm) |
| --- | --- | --- | --- | --- |
| Erebidae | *Arctia caja* | 28.94 | 7.62 | 8.06 |
|  | *Catacola nupta* | 35.6 | 8.37 | 8.21 |
|  | *Catacola promissa* | 26.34 | 6.93 | 6.67 |
|  | *Catacola promissa* | 28.79 | 6.7 | 7.51 |
|  | *Herminia grisealis* | 11.21 | 2.16 | 1.58 |
|  | *Hypena proboscidalis* | 17.35 | 3.37 | 2.86 |
|  | *Lygephila pastinum* | 19.41 | 3.74 | 4.33 |
|  | *Phragmatobia fuliginosa* | 13.01 | 4.3 | 4.45 |
|  | *Phytometra viridaria* | 9.14 | 1.88 | 2.25 |
|  | *Phytometra viridaria* | 8.54 | 1.95 | 2.02 |
|  | *Phytometra viridaria* | 8.44 | 1.66 | 1.87 |
|  | *Tyria jacobaeae* | 16.29 | 3.34 | 3.46 |
|  | *Tyria jacobaeae* | 16.1 | 2.97 | 3.72 |
|  | *Uthesia pulchella* | 15.99 | 2.91 | 2.15 |
|  | *Uthesia pulchella* | 16.4 | 2.96 | 2.4 |
|  | *Uthesia pulchella* | 14.69 | 2.26 | 2.05 |
| Geometridae | *Biston betularia* | 22.41 | 6.13 | 5.5 |
|  | *Biston betularia* | 21.6 | 5.34 | 6.06 |
|  | *Biston betularia* | 23.06 | 5.98 | 6.11 |
|  | *Bupalus piniaria* | 17.99 | 2.64 | 3.27 |
|  | *Bupalus piniaria* | 17.62 | 2.52 | 3.18 |
|  | *Cabera pusaria* | 15.43 | 1.97 | 2.35 |
|  | *Campaea margaritata* | 21.03 | 2.28 | 3.29 |
|  | *Campaea margaritata* | 21.61 | 3.25 | 3.49 |
|  | *Camptogramma bilineata* | 13.72 | 2.09 | 2.8 |
|  | *Camptogramma bilineata* | 12.39 | 2.12 | 2.82 |
|  | *Camptogramma bilineata* | 12.29 | 2.09 | 2.62 |
|  | *Chesias legatella* | 16.98 | 2 | 2.52 |
|  | *Chesias legatella* | 16.88 | 2.34 | 3.05 |
|  | *Chesias legatella* | 17.21 | 2.54 | 3.65 |
|  | *Chiasma clathrata* | 12.46 | 1.88 | 1.7 |
|  | *Ennomos sp.* | 19.67 | 3.56 | 4.48 |
|  | *Hydrelia flammeolaria* | 9.31 | 1.21 | 1.73 |
|  | *Odezia atrata* | 13 | 1.84 | 2.24 |
|  | *Odezia atrata* | 12.36 | 1.94 | 2.15 |
|  | *Odezia atrata* | 14.06 | 2.07 | 2.84 |
|  | *Odontopera bidentata* | 21.38 | 4.66 | 4.95 |
|  | *Odontopera bidentata* | 20.2 | 3.98 | 4.81 |
|  | *Opisthograptis luteolata* | 15.3 | 2.7 | 3.21 |
|  | *Xanthorhoe fluctuata* | 13.47 | 1.98 | 3 |
|  | *Perizoma taeniata* | 10.25 | 1.33 | 2.26 |
| Noctuidae | *Acronicta tridens* | 17.02 | 5.26 | 4.62 |
|  | *Allophyes oxyacanthae* | 17.52 | 3.92 | 5.07 |
|  | *Allophyes oxyacanthae* | 16.56 | 4.78 | 4.69 |
|  | *Allophyes oxyacanthae* | 18.73 | 4.3 | 5.35 |
|  | *Amphipyrea pyramidea* | 22.4 | 6.26 | 6.43 |
|  | *Autographa gamma* | 19.16 | 4.52 | 5.08 |
|  | *Autographa gamma* | 19.22 | 5.02 | 5.19 |
|  | *Autographa gamma* | 19.32 | 5.05 | 4.92 |
|  | *Autographa pulchrina* | 16.87 | 4.5 | 5.01 |
|  | *Autographa pulchrina* | 16.32 | 3.98 | 4.06 |
|  | *Cryphia domestica* | 12.32 | 2.58 | 2.86 |
|  | *Cucullia chamomillae* | 22.88 | 6.36 | 5.35 |
|  | *Noctua janthe* | 17.39 | 4.43 | 4.56 |
|  | *Noctua janthe* | 18.78 | 3.86 | 5.36 |
|  | *Noctua janthe* | 16.92 | 4.76 | 3.84 |
|  | *Noctua pronuba* | 24.56 | 7.48 | 6.87 |
|  | *Noctua pronuba* | 23.94 | 7.64 | 6.64 |
|  | *Noctua pronuba* | 25.02 | 6.61 | 7.25 |
|  | *Phlogophora meticulosa* | 22.86 | 6 | 6.97 |
|  | *Phlogophora meticulosa* | 21.9 | 5.97 | 6.07 |
|  | *Phlogophora meticulosa* | 22.33 | 5.79 | 5.26 |
|  | *Tiliacea citrago* | 14.73 | 3.88 | 3.34 |
|  | *Xanthia citrago* | 16.29 | 3.7 | 3.48 |
|  | *Xanthia icteritia* | 15.37 | 3.82 | 3.93 |

Multiple linear regression in R (R Core Team, 2020) was used to model the relationship between thorax depth and both forewing length and thorax width for these pinned specimens. The predict function was then used to generate values for thorax depth for each specimen within our morphometric trait database. Individual regressions were run at the family-level to provide predictions for thorax depth in the Noctuidae, Geometridae, and Erebidae, and across the total dataset to provide predictions for “other” macro-moth families. In each case, stepwise regression was used to reach a minimal adequate model prior to prediction.

*Lateral and anterior aspect ratios*: We used the predicted values for thorax depth to calculate two aspect ratios: the lateral aspect ratio (body length/thorax depth) and the anterior aspect ratio (thorax width/thorax depth) for each macro-moth specimen within our morphometric trait database.

This left us with a database of 10 measured and derived morphometric traits per individual. From this we calculated the mean value for each trait per species and applied these mean values to the records of macro-moth species abundance per night, creating a corresponding matrix of mean trait values per species per sampling date. A principal component analysis (PCA) of this matrix provided showed that PC1 was broadly related to size and PC2 was broadly related to the aspect ratio of the body (Supplementary Table 1). Raw data for all measurements can be found in Dally et al. (2021).

**Supplementary Table 1.** The variable loadings for the first two principal components (PCs) of the principal component analysis run on the matrix of mean morphometric trait values per species per sampling date. Variables in bold indicate a value of >0.5 (or >-0.5), a baseline used to show that a trait has a strong relationship with a given PC.

| Morphometric trait | PC1 | PC2 |
| --- | --- | --- |
| Mean Forewing Length (mm) | 0.321057 | -0.23067 |
| Mean Body Length (mm) | 0.357148 | -0.01221 |
| Mean Abdomen Length (mm) | 0.337018 | -0.08583 |
| Mean Thorax Length (mm) | 0.343761 | 0.098357 |
| Mean Thorax Width (mm) | 0.347873 | 0.000161 |
| Mean Abdomen Width (mm) | 0.34279 | -0.05276 |
| Mean Thorax Depth (mm) | 0.328399 | -0.37984 |
| Mean Fresh Mass (mg) | 0.32384 | -0.0878 |
| Mean Anterior Aspect Ratio | 0.241693 | **0.522509** |
| Mean Lateral Aspect Ratio | 0.167214 | **0.708384** |

**Supplementary References**

Dally, T., Evans, W.L. and Jane, J. (2021) Morphometric measurements relating to 177 macro-moth species collected in the UK, 2017. NERC Environmental Information Data Centre, https://catalogue.ceh.ac.uk/documents/3a813bd9-59ae-476e-a170-ab83fe1587b2

García-Barros, E. (2015) Multivariate indices as estimates of dry body weight for comparative study of body size in Lepidoptera. *Nota Lepidopterologica,* 38(1): 59-74. DOI: <https://doi.org/10.3897/nl.38.8957>

[Kinsella](https://onlinelibrary.wiley.com/action/doSearch?ContribAuthorStored=Kinsella%2C+Rebecca+S) R. S., Thomas, C. D., Crawford, T. J., Hill, J. K., Mayhew, P. J., and MacGregor, C. J. (2020). Unlocking the potential of historical abundance datasets to study biomass change in flying insects. *Ecology and Evolution.* DOI: 10.1002/ece3.6546

Natural History Museum (2014). Dataset: Collection specimens. Natural History Museum Data Portal (data.nhm.ac.uk). DOI: <https://doi.org/10.5519/0002965>

Natural History Museum (2018). NHMUK010492103. Natural History Museum Data Portal (data.nhm.ac.uk). [Online]. [Accessed 17^th^ June 2021]. Available from: https://data.nhm.ac.uk/object/2f899129-e549-4191-8fd4-5d9b2dfe001c/1623888000000

R Core Team (2020). R: A language and environment for statistical computing. *R Foundation for Statistical Computing*, Vienna, Austria. URL: <https://www.R-project.org/>.

Rydell, J., and Lancaster, W. C. (2000). Flight and thermoregulation in moths were shaped by predation from bats. *OIKOS,* 88: 13–18. DOI: <https://doi.org/10.1034/j.1600-0706.2000.880103.x>

Schneider, C., Rasband, W., and Eliceiri, K. (2012). NIH Image to ImageJ: 25 years of image analysis. *Nat. Methods.,* 9: 671–675. DOI: https://doi.org/10.1038/nmeth.2089

Skinner, B. (2009). Colour Identification Guide to the Moths of the British Isles. 3rd ed. Stenstrup, Denmark: Apollo Books.
